# Uncovering floral composition of paper wasp nests (Hymenoptera: Vespidae: Polistes) through DNA metabarcoding

**DOI:** 10.1038/s41598-024-52834-6

**Published:** 2024-02-03

**Authors:** Saeed Mohamadzade Namin, Minwoong Son, Chuleui Jung

**Affiliations:** 1 Agricultural Science and Technology Institute, Andong National University, Andong, Republic of Korea; 2 Rural Development Administration (RDA), Jeonju, Republic of Korea; 3 Department of Plant Medicals, Andong National University, Andong, Republic of Korea

**Keywords:** Entomology, Community ecology, Molecular ecology

## Abstract

As the social organism, *Polistes* wasps build a communal nest using woody fibers with saliva for sustaining brood and adult population throughout the season. Limited information exists regarding the identification specific plant materials employed in wasp nest building. Thus, we firstly tested if the DNA metabarcoding approach utilizing *rbcL* and *trnL* molecular markers could identify the plant species quantitatively and qualitatively inform the mixed-origin woody samples. A threshold of 0.01 proportion of reads was applied for *rbcL* and *trnL* molecular markers, while this threshold for median proportion was 0.0025. In assessing taxa richness, the median proportion demonstrated superior performance, exhibiting higher taxa detection power, however, *rbcL* marker outperformed in quantitative analysis. Subsequently, we applied DNA metabarcoding to identify the plant materials from the nests of two *Polistes* species, *P. mandarinus* and *P. rothneyi.* The results showed that higher preference of *Quercus* and *Robinia* as the major nest building materials regardless of the surrounding plant communities, by two wasp species. Material diversity was higher for *P. rothneyi* than *P. mandarinus*, which may explain the abundance of this species possibly with heightened adaptive capacities in their nesting behavior. This study demonstrated that DNA metabarcoding could identify the complex nest-building plant materials of paper wasps and provide insights into their ecological interactions in the natural ecosystem.

## Introduction

The genus *Polistes* comprising approximately 200 species is widely distributed in tropical and temperate regions of the world^1^. These predatory insects primarily prey on caterpillars^2–4^ and construct unenclosed paper nests characterized by an umbrella-shape structure that includes hexagonal cells suspended with one or more pedicels from shrubs, herbaceous plants, or structures such as window frames and roofs in urban areas^5–7^. The nests serve as a place for rearing brood in eusocial wasps by providing a shelter for the protection of immature stages against natural enemies and temperature extremes^8,9^. During early summer, the foundress of a *Polistes* searches for the best location to make a nest and starts building a pedicel of the nest by macerated wood and plant fibers^10^. In the newly established nests, foraging activities are primarily related to nest construction material, while the consumption of arthropod pray gradually increases in comparison with wood pulp consumption through the season, as only nest repair activities become necessary^11,12^.

Workers in *Polistes* nests perform a variety of tasks, including cleaning, feeding larvae, defense, and foraging for nectar, hunting or nest-building materials such as wood pulp and water^13,14^. Nest construction in social wasps is a complex activity requiring a combination of three tasks: water foraging, pulp foraging, and building^15^. In larger colonies of social wasps, with a greater number of workers, task division related to nest construction is observed, while in smaller colonies, frequent switching of the task occurs in generalist workers, and the pulp foragers switch to builders after collecting and transferring the pulp into the nest^15–17^. Foragers generally tend to switch to another functionally related foraging task rather than switching between building materials to food materials^18,19^.

Different materials such as paper or filter papers have been provided for paper wasps in the laboratory condition to investigate the biology, nest structure, and nest-making behavior of these social insects^20,21^. Limited research has been conducted on wasps' wood collection behavior, but it has been observed that foragers use wood fiber from different sources, including sound or weathered wood from fences, posts, dead trees, decayed wood, bark of living trees and shrubs, cortex of non-woody plants, and other papery materials such as paper, hard paper, and clothing in their nest construction^22^. However, knowledge about the specific type of wood pulp resources used by wasps is limited. Several studies have documented the collection of wood pulp by *Vespa crabro* from the rotten wood and inner bark of dead and living trees, such as lilac and ash^23–25^. Bronly^26^ reported that birch, *Rhododendron* and *Dahlias* are also sources of cortical material in wasp nests. *P. germanica* has been observed collecting weathered wood from chestnut paling and soft-wood fences and posts^22^, while *Dolichovespula norwegica* has been reported to gather woolly pubescence from *Rhododendron* leaves^27^. Recently, Khan et al.^28^ conducted a multiple-choice test using woods from five different woody trees to investigate the wood preferences of *P. flavus* in nest construction. The results showed a preference for *Populus deltoid* over the other options. Overall, while some information is available regarding wasps' wood collection behavior, more research is needed to fully understand their preferences and the specific types of wood pulp resources used by different species.

Monitoring the foraging activities of *Polistes* species is challenging due to the availability of numerous workers inside each nest and their flight ability of more than 150 m^29–31^. Recently, molecular approaches such as DNA barcoding and metabarcoding have been used to identify the prey diversity of *Polistes* species in different landscapes^3,4,32^. It is crucial to have knowledge of the preferred wood for nest-making in laboratory studies to mimic natural conditions and to study possible habitats where paper wasps can occupy in nature currently, and to predict their future distribution in response to global warming and climate change. The main purpose of this study is to evaluate the possibility of applying DNA metabarcoding to identify the floral composition of wasp nest. We analyzed two different molecular markers as well as median proportion to select the best molecular marker in wasp nest analysis. Then, we determined the plant composition of nest from two different species of paper wasps, *P. mandarinus* (with yellowish brown nest) and *P. rothneyi* (dark gray nest) to address whether different wasps use different resources for their nest-making. Answering this question can lead us to discover the evolution of nest-making behavior in different groups of social wasps.

## Results

### Analysis of bark mock communities

#### Taxonomic identification, false negative and false positive taxa

We conducted an experiment to assess the feasibility of using DNA metabarcoding to detect plant taxa within mixed wooden structures such as wasp nests. We prepared seven artificial bark mixtures with different proportions of nine different plant species (Table 1). In all of the bark mixtures, DNA metabarcoding was able to detect all of the species present. When using the *rbcL* marker, *Rhus chinensis* was classified as family Anacardiaceae, and four species could only be identified to the genus level (*Pinus*, *Populus*, *Zanthoxylum* and *Paulownia*), while three species were identified to the species level (*Ailanthus latissimus*, *Robinia pseudoacacia*, *Lindera obtusiloba*). *Prunus salicina* was recognized as *Prunus* sp. in two samples, but was misidentified as *Prunus mume* in PC7. When using *trnL* marker, reads assigned to *Robinia pseudoacacia* were classified as family Fabaceae, and five species were identified to the genus level (*Pinus*, *Paulownia*, *Populus*, *Prunus*, *Zanthoxylum*), while three species were identified to the species level (*L. obtusiloba*, *R. chinensis* and *A. latissimus*). Both molecular markers were able to identify five taxa to the genus level (*Pinus*, *Populus*, *Zanthoxylum* and *Paulownia*), and two species to the species level (*L. obtusiloba*, and *A. latissimus*). In the median analysis, a consensus taxonomy identification from both markers was used, resulting in the recognition of five taxa to the genus level and four taxa to the species level. However, *Prunus mume* was misidentified into species level in one sample.

**Table 1:** The real proportion of mixed bark samples and related proportion of *trnL*, *rbcL* and the median proportion. The median proportion is the average of *rbcL* and *trnL* proportions.

Sample	Taxa	Real proportion	*trnL*		*rbcL*		Median	
			Taxa ID	Proportion	Taxa ID	Proportion	Taxa ID	Proportion
PC1	*Robinia pseudoacacia*	0.4	Fabaceae	0.813	*Robinia pseudoacacia*	0.958	*Robinia pseudoacacia*	0.886
	*Prunus salicina*	0.6	*Prunus*	0.187	*Prunus*	0.042	*Prunus*	0.114
PC2	*Pinus koreanus*	0.78	*Pinus*	0.480	*Pinus*	0.620	*Pinus*	0.550
	*Populus tomentiglandulosa*	0.22	*Populus*	0.520	*Populus*	0.380	*Populus*	0.450
PC3	*Populus tomentiglandulosa*	0.55	*Populus*	0.458	*Populus*	0.874	*Populus*	0.666
	*Robinia pseudoacacia*	0.16	Fabaceae	0.393	*Robinia pseudoacacia*	0.100	*Robinia pseudoacacia*	0.247
	*Rhus chinensis*	0.27	*Rhus chinensis*	0.149	Anacardiaceae	0.026	*Rhus chinensis*	0.088
PC4	*Zanthoxylum schinifolium*	0.34	*Zanthoxylum*	0.499	*Zanthoxylum*	0.453	*Zanthoxylum*	0.476
	*Lindera obtusiloba*	0.33	*Lindera obtusiloba*	0.366	*Lindera obtusiloba*	0.420	*Lindera obtusiloba*	0.393
	*Prunus salicina*	0.33	*Prunus*	0.135	*Prunus*	0.108	*Prunus*	0.121
PC5	*Pinus koreanus*	0.27	*Pinus*	0.507	*Pinus*	0.504	*Pinus*	0.505
	*Ailanthus altissima*	0.22	*Ailanthus altissima*	0.393	*Ailanthus altissima*	0.146	*Ailanthus altissima*	0.269
	*Paulownia tomentosa*	0.4	*Paulownia*	0.012	*Paulownia*	0.314	*Paulownia*	0.163
	*Lindera obtusiloba*	0.08	*Lindera obtusiloba*	0.031	*Lindera obtusiloba*	0.033	*Lindera obtusiloba*	0.032
	*Rhus chinensis*	0.03	*Rhus chinensis*	0.057	Anacardiaceae	0.004(0)	*Rhus chinensis*	0.030
PC6	*Populus tomentiglandulosa*	0.24	*Populus*	0.425	*Populus*	0.310	*Populus*	0.368
	*Paulownia tomentosa*	0.02	*Paulownia*	0.002(0)	*Paulownia*	0.003(0)	*Paulownia*	0.003
	*Rhus chinensis*	0.01	*Rhus chinensis*	0.001(0)	Anacardiaceae	0.000(0)	*Rhus chinensis*	0.001(0)
	*Zanthoxylum schinifolium*	0.17	*Zanthoxylum*	0.079	*Zanthoxylum*	0.021	*Zanthoxylum*	0.050
	*Pinus koreanus*	0.46	*Pinus*	0.390	*Pinus*	0.647	*Pinus*	0.519
	*Ailanthus altissima*	0.07	*Ailanthus altissima*	0.097	*Ailanthus altissima*	0.017	*Ailanthus altissima*	0.057
	*Lindera obtusiloba*	0.03	*Lindera obtusiloba*	0.005(0)	*Lindera obtusiloba*	0.001(0)	*Lindera obtusiloba*	0.003
PC7	*Lindera obtusiloba*	0.005	*Lindera obtusiloba*	0.000(0)	*Lindera obtusiloba*	0.000(0)	*Lindera obtusiloba*	0.000(0)
	*Pinus koreanus*	0.24	*Pinus*	0.460	*Pinus*	0.226	*Pinus*	0.343
	*Populus tomentiglandulosa*	0.5	*Populus*	0.430	*Populus*	0.759	*Populus*	0.595
	*Paulownia tomentosa*	0.1	*Paulownia*	0.004(0)	*Paulownia*	0.004(0)	*Paulownia*	0.004
	*Rhus chinensis*	0.05	*Rhus chinensis*	0.042	Anacardiaceae	0.002(0)	*Rhus chinensis*	0.022(0)
	*Zanthoxylum schinifolium*	0.01	*Zanthoxylum*	0.006(0)	*Zanthoxylum*	0.001(0)	*Zanthoxylum*	0.003
	*Prunus salicina*	0.07	*Prunus*	0.001(0)	*Prunus mume*	0.003(0)	*Prunus mume*	0.002(0)
	*Robinia pseudoacacia*	0.005	Fabaceae	0.023	*Robinia pseudoacacia*	0.003(0)	*Robinia pseudoacacia*	0.013
	*Ailanthus altissima*	0.02	*Ailanthus altissima*	0.035	*Ailanthus altissima*	0.001	*Ailanthus altissima*	0.018

The negative controls were also included in every stage of the DNA extraction, library preparation, and sequencing processes. The sequencing of the negative control using *rbcL* gene did not yield any reads (Supplementary Table 2), while the number of reads in the negative control after quality control trimming was 3098 for *trnL* (Supplementary Table 1). In the negative control, ten taxa (*Alnus*, *Lindera obtusiloba*, *Fabaceae*, *Poaceae*, *Quercus*, *Pinus*, *Glynce*, *Diospyros*, *Prunus*, *Solanum*) were observed, most of which were used in the bark mixture preparation, and the number of reads for each taxa was used as a threshold for removing rare taxa among bark mixtures. All statistics and calculations based on the *trnL* marker were applied after removing the reads belonging the taxa present in negative control. In addition to the negative control, *Rhus*, *Alnus*, *Paulownia*, *Ailanthus*, *Ulmus*, *Prunus*, *Achillea millefolium Populus*, *Pinus*, *Diospyros*, *Glynce*, Salicaceae, *Albizia*, *Robinia* were detected as a false positive in seven bark mixtures using *trnL* marker while *Populus*, *Diospyros*, *Pinus*, *Quercus*, *Brassica*, *Litsea*, *Alnus*, and *Lindera* were found as a false positives using *rbcL* marker (Supplementary Table 4). Except for PC4, where *Litsea* covered 0.009 of *rbcL* reads and *Populus* covered 0.01 proportion of *trnL* reads, the highest proportion of the false positive taxa among other PC samples was 0.003 for both markers. False positive taxa were only detectable with either *rbcL* or *trnL* markers, except for PC1, where *Populus*, *Pinus* and *Diospyros* were detectable in very low abundances using both markers (0.0015–0.0025 in *trnL* and 0.0012–0.0022 in *rbcL*) (Supplementary Table 4).

#### Quantitative matching of sequence reads to the bark mixtures

**Figure 1:**
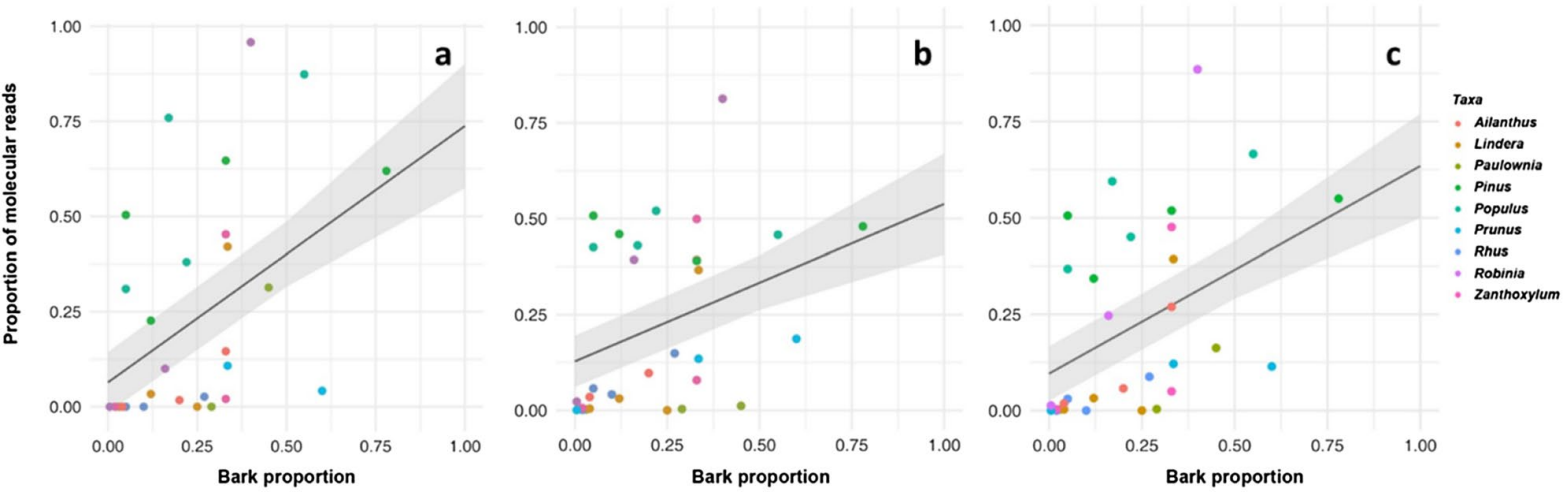
The correlation between the observed proportion of each taxon with the real proportion of the same taxon in seven bark mixtures using linear mixed effect model (LMEM) for *rbcL* (**a**), *trnL* (**b**) and median proportion (**c**).

We conducted a LMEM analysis to assess the quantitative matching between the sequence reads and the actual proportion of taxa in the bark mixtures. Our results indicated a positive correlation between the proportion of *rbcL* and *trnL* reads and the true proportion of taxa. We also calculated the median proportion of the two molecular markers, which demonstrated a significant correlation with the actual proportion of species in the bark mixtures. The *rbcL* marker showed the strongest correlation (Adjusted R2: 0.54, intercept = 0.065, slope = 0.67), followed by the median correlation (Adj. R2 = 0.59, intercept = 0.096, slope = 0.53) and *trnL* reads (Adj. R2 = 0.53, intercept = 0.12, slope = 0.41) (Fig. 1, Supplementary Table 3). Based on our analysis of the PC samples using LMEM, we determined that the proportion of reads based on the *rbcL* marker is more reliable for quantitative analysis. Therefore, we proceeded with the next analysis using the abundances observed using the *rbcL* marker. However, to avoid potential false positives, we removed all taxa with a relative proportion lower than 0.01 from the list of taxa.

### Analysis of the plant composition of Polistes nest

#### Floral composition of Polistes nest

**Figure 2:**
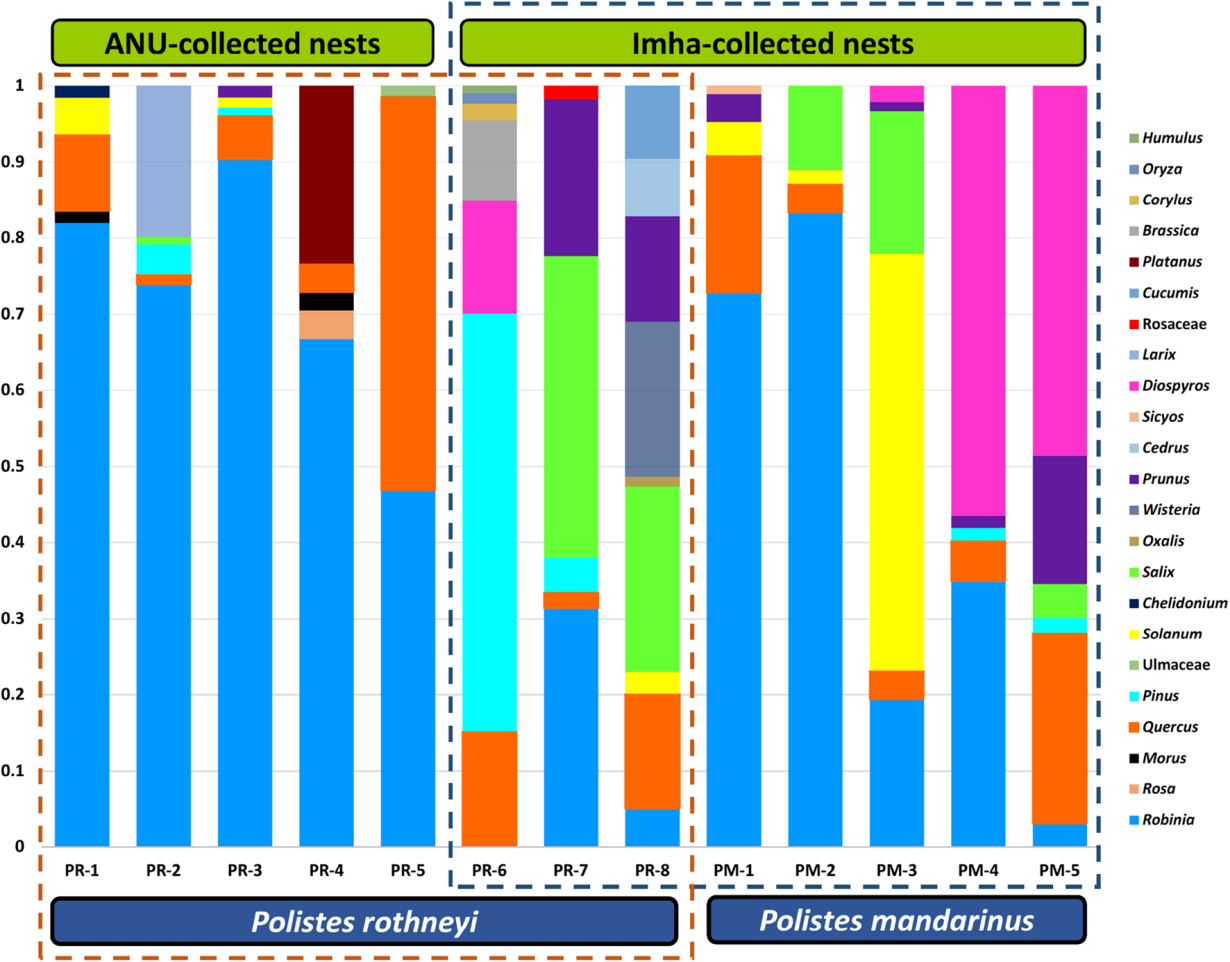
Bar charts showing the proportion of taxonomic units at genus level in the nest samples of *P. mandarinus* (PM-n) and *P. rothneyi* (PR-n). The top bar shows the locality of nest collection, while the bottom bar indicates the taxonomic identification of the nest materials.

The plant composition of 13 collected nests belonging to *P. mandarinus* and *P. rothneyi* was analyzed using sequence reads of the *rbcL* marker. To prevent false positives, only plant taxa with a proportion higher than 0.01 were selected for further analysis. In total, 23 plant taxa were identified. Seven plant taxa (*Robinia*, *Pinus*, *Prunus*, *Quercus*, *Solanum*, *Salix*, and *Diospyros*) were found in nests of both species (Figs. 2, 5).

**Figure 3:**
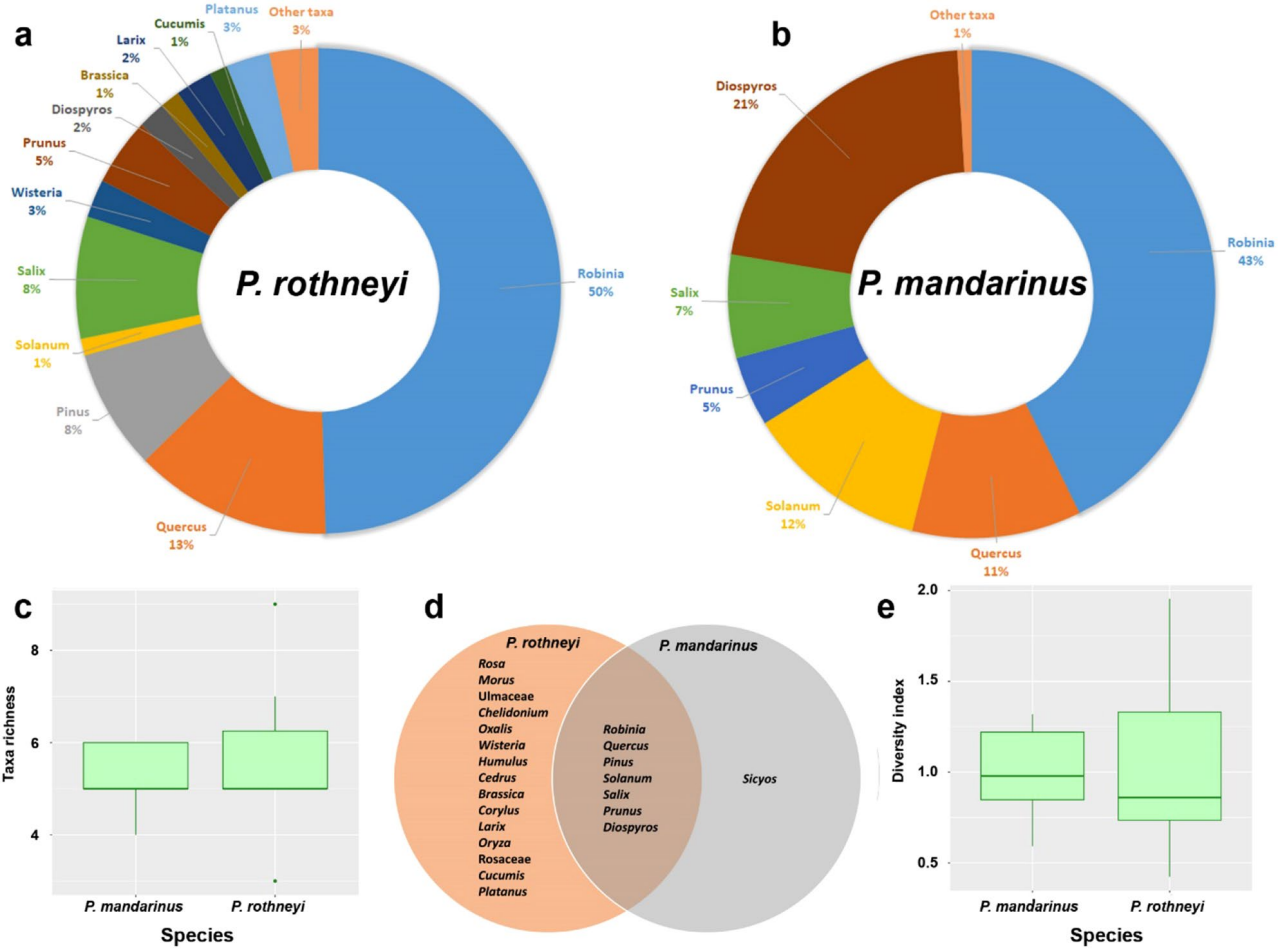
(**a**, **b**) Pie charts showing the taxonomic composition of major plant taxa (> 1% abundance) at the genus level in *P. rothneyi* nests and *P. mandarinus* nests. The abundances of taxa are reported as the average proportion of that taxa in the all samples from the same species. Taxa accounting for < 1% of reads are grouped as “Other taxa”. (**c**) Boxplots of the Shannon diversity index among the nests of *P. rothneyi* and *P. mandarinus*. (**d**) Venn diagram showing the number of unique and shared plant taxa identified at the genus level among nest samples. (**e**) Boxplots of the taxa richness among the nests of *P. rothneyi* and *P. mandarinus*.

*Quercus* was the only plant taxa that was found in all nests belonging to *P. mandarinus* and *P. rothneyi* indicating its importance as a primary plant for nest construction*. Robinia* was the second most abundant taxa and was only absent in one of the PRNs. *Prunus*, *Pinus*, *Salix* and *Solanum* were also highly abundant taxa found in approximately half of *Polistes* nests. Except *Diospyrus* which was found in three *P. mandarinus* and one *P. rothneyi* nest, and *Morus* which was found in two *P. rothneyi* nest, the other sixteen taxa were present in only one nest, and among them, only *Sicyos* was found in a PMN, while the other fifteen were exclusively found in PRNs (Fig. 3d. Interestingly, the seven most abundant taxa found in PMNs were also present in at least one of the PRNs (Figs. 2, 5). *Diospyros* was only available in the nest collected in Imha district. Additionally, *Prunus* and *Salix* were found in six and five nests of Imha district, respectively, but were only present in one nest from ANU.

Comparing the total proportion revealed that *Robinia* was the most abundant taxa in both groups of nests, accounting for 50% and 43% of the total floral composition in PRNs and PMNs, respectively (Fig. 3a,b). In PMN, *Diospyros*, *Solanum*, *Quercus*, *Salix,* and *Prunus* were the most dominant plant taxa, each representing more than 5% of the total floral composition, while *Quercus*, *Pinus*, *Salix,* and *Prunus* were the major taxa present in PRNs (Fig. 3a,b). Taxa richness in PRNs varied between 3 and 9 compared to the 4–6 taxa observed in PMNs, but the difference between them was not significant (Mann–Whitney U test, W = 18, *p* = 0.816) (Fig. 3c). In addition, no significant differences were observed when comparing the taxa diversity between PRNs and PMNs (Mann–Whitney U test, W = 20, *p* = 1) (Fig. 3e).

#### Comparing *P. rothneyi* nests form two different localities

**Figure 4:**
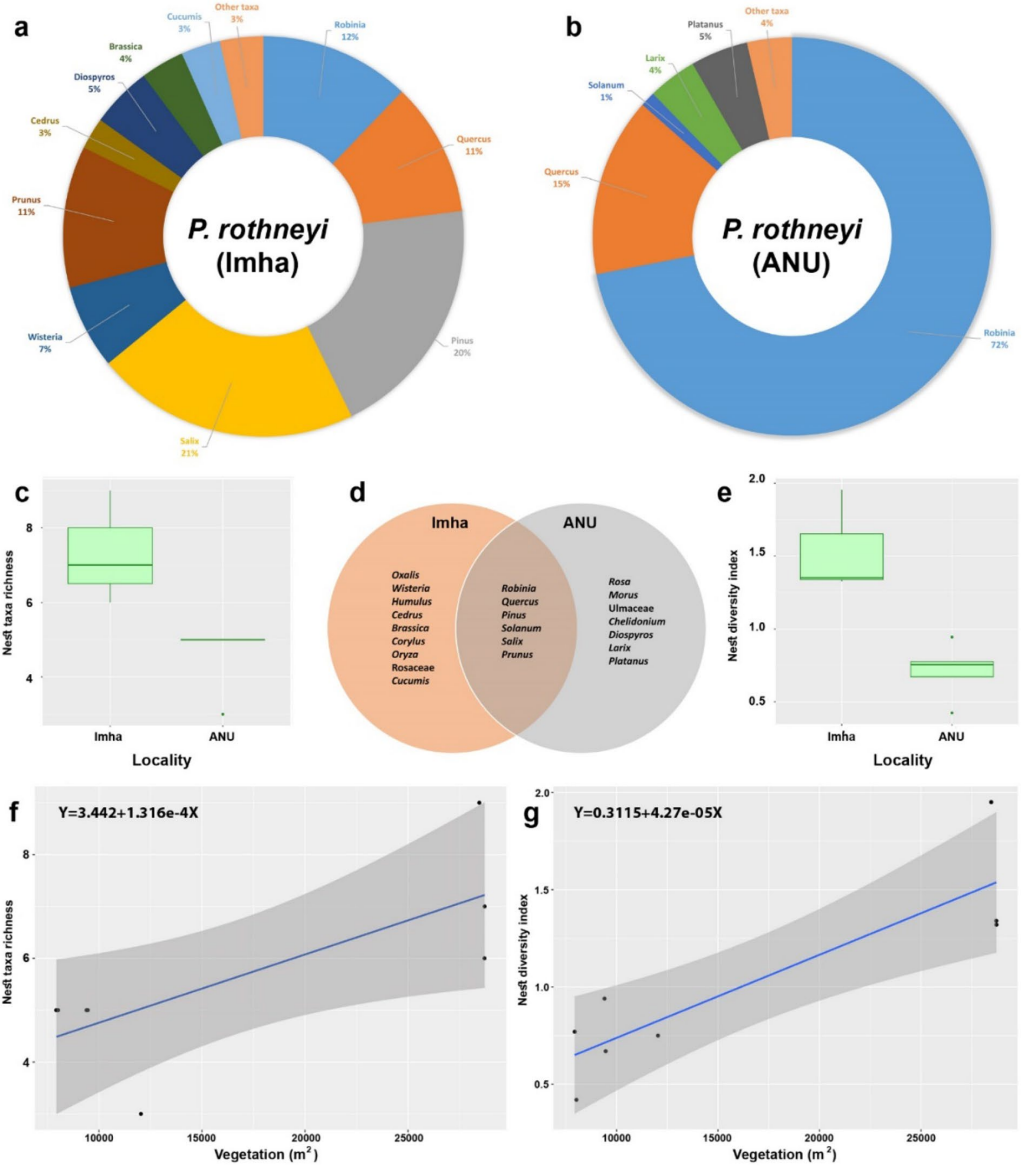
(**a**, **b**) Pie charts showing the taxonomic composition of major plants (> 1%) at genus level in *P. rothneyi* nests collected from Imha district (**a**) and ANU (**b**). Abundances of taxa are reported with the average proportion of that taxa in the all samples collected from the same locality. Taxa accounting for < 1% of reads are grouped as “Other taxa”. (**c**) Boxplots of the Shannon diversity index among the nests of *P. rothneyi* collected from Imha district and ANU. (**d**) Venn diagram shows the number of unique and shared taxa identified at the genus level among nest samples from different localities. (**e**) Boxplots of the taxa richness among the nests of *P. rothneyi* collected from Imha district and ANU. (**f**) Relationship between the taxa richness per nest and the vegetation cover 100m radius around the nest (**g**). Relationship between the taxa diversity per nest and the vegetation cover 100m radius around the nest. See Supplementary Table 8 for model output.

Since the PRNs were collected from two different localities, understanding the floral preference of this species in different locations with varying floral diversity and land use provides more information about their nest-making behavior and wood collection foraging activities. Comparing the total floral composition between five nests collected at ANU and three nests collected at Imha district indicated a significant difference in floral composition and diversity. In the nests collected at ANU, *Robinia* was the most dominant taxon with a relative proportion of 72%, followed by *Quercus* (15%) and *Platanus* (5%). On the other hand, *Salix* (21%) and *Pinus* (20%) ware the most dominant taxa among PRNs form Imha district, followed by *Robinia*, *Quercus*, *Prunus*, *Wisteria* and *Diospyros* with relative abundances of 7–12%. Furthermore, *Robinia* and *Quercus* were found in all ANU-collected nests (Fig. 4a), while *Quercus* and *Pinus* were present in all Imha-collected nests (Fig. 4b). More importantly, the taxa richness varied between 3 and 5 in ANU-collected PRNs, while the taxa richness in Imha-collected nests varied from 6 to 9, showing significantly higher taxa richness among Imha-collected PRNs (Mann–Whitney U test, W = 15, *P* value = 0.0261) (Fig. 4c). In addition, the Shannon diversity index of the PRNs from Imha (1.32–1.95) was significantly higher than that of the nests from ANU (0.43–0.94) (Mann–Whitney U test, W = 15, *P* value = 0.037) (Fig. 4e). *Robinia*, *Quercus*, *Pinus*, *Solanum*, *Salix* and *Prunus* were present in at least one nest collected from both localities, while several taxa only found to be present in the nests collected form only one locality (Fig. 4d). Furthermore, the regression analysis showed that the correlation between the taxa richness and vegetation area in the foraging range across PRNs was statistically significant (R2 = 0.559, *p* < 0.05). Additionally, significantly positive correlation was observed between taxa diversity and the vegetation area (R2 = 0.766, *p* < 0.01), with larger vegetation associated with higher number of detected taxa.

#### Correlation between the nests samples

**Figure 5:**
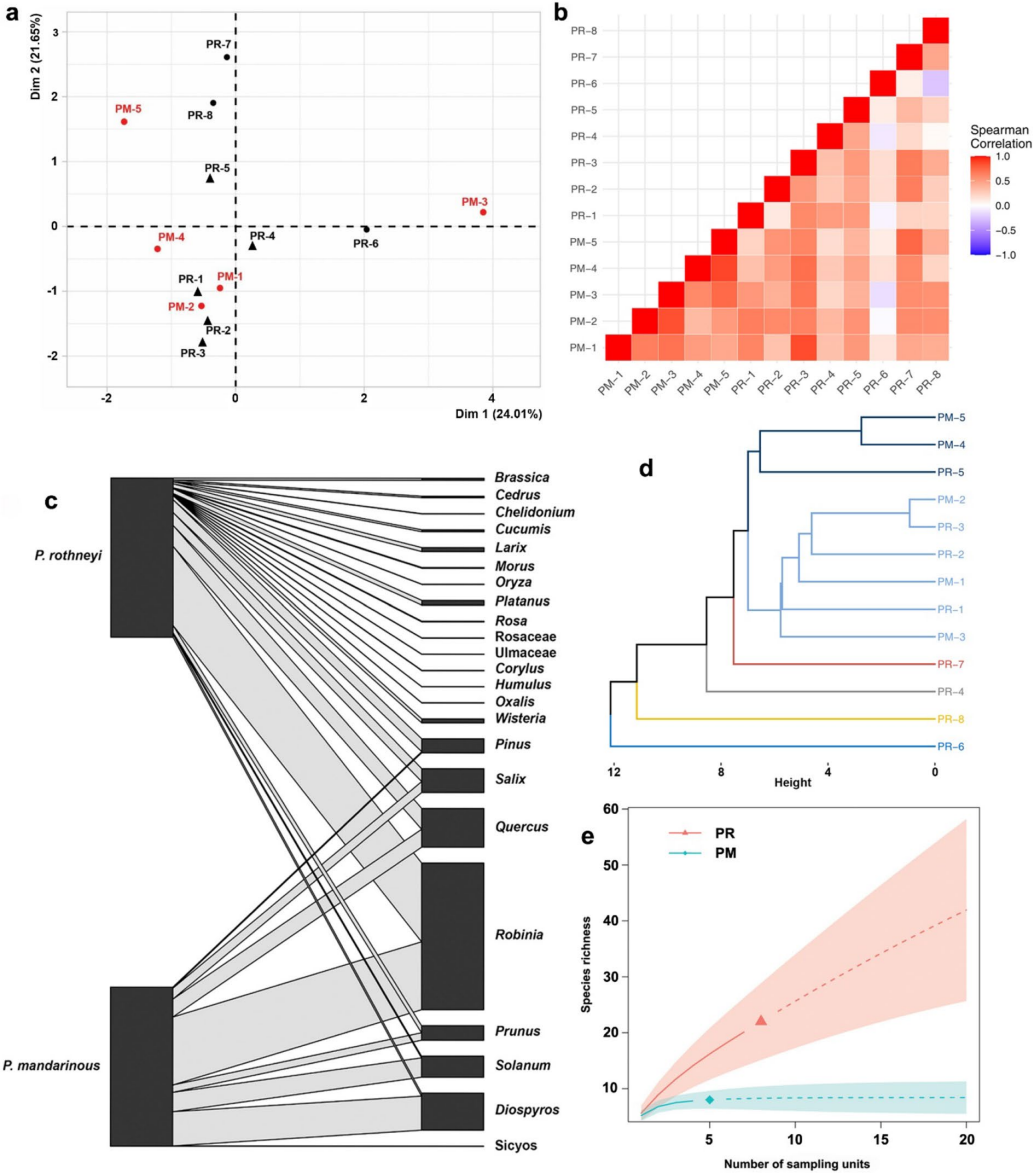
(**a**) Correspondence analysis (CA) representing the plant taxa and their abundances in the nest samples from *P. mandarinus* (PM-n) and *P. rothneyi* (PR-n). (**b**) A heat map representation of the Spearman correlation matrix of the nest samples based on their floral composition created using ggplot2 package in R. (**c**) Bipartite graphs of the interaction between wasp species with pulp foraging plants. Left bars represent wasp species, *P. mandarinus* and *P. rothneyi* and right bars represent plant taxa. The linkage width indicates the frequency of reads from each plant taxa in nests collected from a given wasp species in this study. (**d**) The Euclidean distance–based dendrogram summarizes the relationship among nest samples from *P. rothneyi* and *P. mandarinus*. (**e**) Rarefaction-based plant species accumulation curves for *P. rothneyi* (PR) and *P. mandarinus* (PM) nest samples.

Intraspecific pairwise comparisons of the nest samples showed a significant (*p* < 0.05) correlation in the abundance of plant taxonomic units in PMNs. However, correlations between PRN samples were complicate and were not always significant. Even negative correlations were observed between Imha-collected samples, PR6 and PR8 (Fig. 5b). In interspecific pairwise comparisons, a positive significant correlation was observed between PR7 (Imha) and PR3 (ANU) with all PMN samples. No significant correlation was found between PR6 and any other PRN or PMN samples (Fig. 5b). Spearman’s Rho ranged from 0.36 to 0.88 in PMN and from − 0.247 to 0.66 in PRN (0.12–0.6 for ANU-collected samples and − 0.247–0.46 for Imha-collected samples).

The correspondence analysis (CA) results demonstrated that major plant taxa used for nest making by the two different wasps were not species-specific and the nest samples from different species did not make any clear cluster (Fig. 5a). The hierarchical cluster analysis showed a stronger relationship between PMNs from the Imha region and ANU-collected PRNs while Imha-collected PRN samples were distantly related with the other nest samples from both species (Figs. 5 d).

#### Correlation between the nests floral composition and surrounding landscape flora

The correlation between the proportion of plants observed in each nest was compared with the floral composition available in a 100-m radius landscape surrounding each nest. The objective was to understand the level of selectivity and adaptation exhibited by two wasp species in relation to the landscape. Positive correlation was observed (except one PR-6) between the floral composition of nests of both species with floral composition of woody plants in surrounding landscape (R square = 0.18–0.35 in PMNs and − 0.02–0.31 in PRNs). No significant difference was found between the R square value of the nest correlation of these two species (Mann–Whitney U test, W = 25, *P* value = 0.509) (Supplementary Fig. 2).

**Figure 6:**
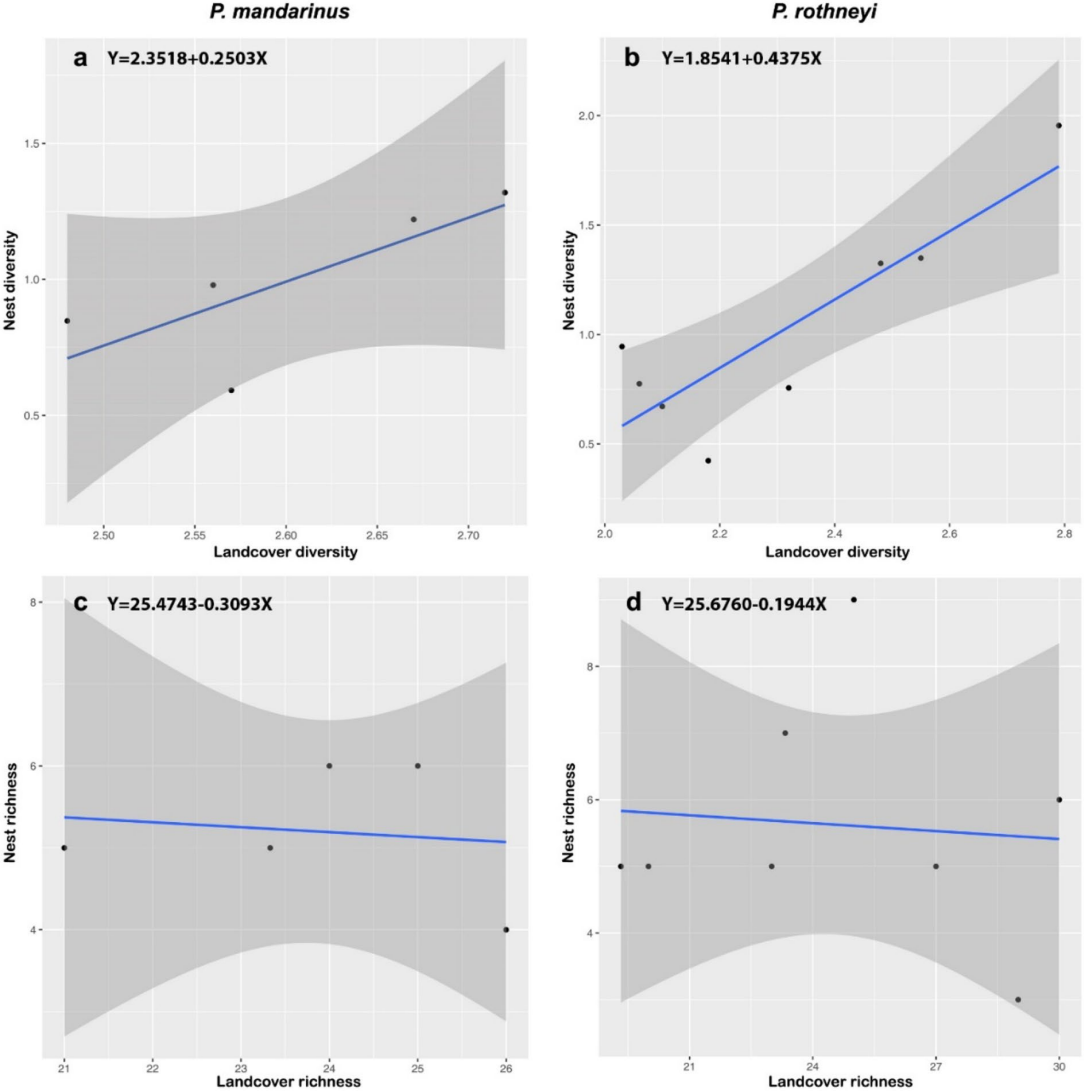
(**a**, **b**) Relationship between the taxa diversity per nest and the taxa diversity of the 100m radius landscape around each nest in PMNs (**a**) and PRNs (**b**); (**c**–**d**) relationship between the taxa richness per nest and the estimated taxa richness of the 100m radius landscape around nest in PMNs (**c**) and PRNs (**d**). See Supplementary Tables 10–11 for model output.

Additionally, the regression analysis revealed a statistically significant positive correlation between the taxa diversity of PRNs and PMNs and the flora taxa diversity in the surrounding landscape (Fig. 6a,b). However, no correlation was observed when comparing the taxa richness of the nest samples with the estimated taxa richness on surrounding landscape (Fig. 6c,d).

## Discussion

### Evaluation of molecular markers for floral composition analysis of wasp nest

DNA metabarcoding is a powerful and time-saving method that is being increasingly used to study the foraging behavior of insects through analysis of floral composition of pollen loads and honey, plant-pollinator interactions, analysis of airborne pollen, and forensic studies^33–35^. In addition, this method allows for the detection of a wide range of plant taxa, including those that may be difficult to identify using traditional methods such as visual inspection or microscopic analysis. In this study, we evaluated the possibility of using DNA metabarcoding to analyze the floral composition of wasp nests. We compared two different molecular markers as well as median proportion. Sequencing of *trnL* marker resulted in higher number of reads, which was expected given its shorter length (10–143 bp for *trnL* in comparison with ~ 500 bp for *rbcL* marker). In addition, the significantly higher rate of high-quality reads in *trnL* can be attributed to the lower level of DNA degradation due to its smaller fragment of target DNA. Airborne pollen trapped in cracks available on the surface of tree barks can be a possible source of false positives in our studies. The higher rate of false positives when using the *trnL* marker can be related to the better detection power of small fragment markers, which can be applied in old and degraded samples. All plants used in artificial mixtures were detectable using both molecular markers; however, after setting the removal thresholds based on the negative control and the available false positives, some plant taxa were not detectable. False negatives observed in three samples using the *rbcL* marker and in two samples when *trnL* and median calculation were applied. The highest false negatives were observed in PC7 using *rbcL* marker, where only three out of nine taxa was detectable. However, in the same sample, only four and three false negatives were observed using *trnL* and median calculation, respectively. Despite some missing taxa using *rbcL* marker, LMEM analysis showed it is more reliable than *trnL* and the median proportion in quantitative studies.

There is ongoing discussion about the feasibility of using molecular markers for quantitative analysis, and some studies have shown that DNA metabarcoding is better suited for present/absent analysis rather than quantitative calculations^36^. While the ITS2 marker has been proposed as a suitable molecular marker for quantitative analysis^37^, many researchers believe that plastid markers such as *rbcL*, *trnL*, and *matK*^38–41^ are more reliable for quantitative analysis.

### Analysis of the plant composition of Polistes nest

We used DNA metabarcoding to identify the plant species used in nest construction by two *Polistes* wasp species. Most of the plant taxa detected in the nests were woody plants, which is consistent with recent microscopic analysis showing the use of both softwood and hardwood forest species, as well as leaves and small particles of agricultural origin in nest construction^42^. *Quercus* and *Robinia* were found to be the most commonly used plants for nest construction by both *Polistes* species. This information could have important implications for conservation efforts, as these plant species may be important for the survival of the wasps. *Quercus* was detected from all wasp nests, while *Robinia* was the most abundant plant taxon, accounting for almost half of the total sequence reads in both PRNs and PMNs. *Robinia* is an introduced taxon in South Korea, and the preference for *Robinia* as a nesting material by these native wasp species could be due to its favorable characteristics, such as ease of excavation and manipulation, which may facilitate nest construction. The softness of the stem might allow the wasps to create nest structures with greater ease compared to other woody plants with harder or more rigid stems. By understanding the specific plant preferences of these wasp species, particularly the attraction to introduced taxa like *Robinia*, we can gain valuable insights into their nesting behaviors and foraging strategies. Although the multiple-choice test analysis by Khan et al.^28^ showed that *P. flavus* preferred *Populus deltoid* over other woody plants, none of the highly abundant taxa in our study were on the list of offering plants. It would be interesting to test these plant taxa in another multiple-choice experiment. We also found several herbaceous plants in the wasp nests, with most of the herbaceous plant taxa detected in the nests being present at very low proportions. However, *Brassica* was present at a proportion of 0.1 in one sample, and *Solanum* was the most abundant taxon in one nest. Although most *Solanum* species are herbaceous, its high abundance in our study may be attributed to the presence of some vine species such as *S. dulcamara* in the study area which was not detectable in our landscape flora essay. We also observed a very small field of *Solanum tomentosum* located in 130m southern part of the nest collection area in Imha district and the presence of *Solanum* can be also attributed to the collection of nest material on the dry post-harvest leftover parts of plants.

With the exception of *Sicyos*, all identified plant taxa present in PMNs were shared with PRNs, underscoring the wide range of plant resources that *P. rothneyi* incorporates into its nest construction (Fig. 5c,e). In the context of social wasps, the collection of plant pulps and water typically occurs in proximity to the nest, contrasting with the foraging patterns for nectar or protein. This phenomenon results in a significantly shorter time allocated to pulp foraging compared to the food collection^2,43,44^. In the analysis solely focusing on Imha-collected PRNs in comparison with PMNs, notable distinction emerged, highlighting the higher taxa diversity and relatively elevated taxa richness within PRNs. The absence of *Robinia* in one PRN sample, high exclusive interaction of PRNs in both localities (Fig. 1), as well as the positive correlation between taxa richness and diversity with the vegetation area (Fig. 4f,g), and high correlation of nest taxa diversity within PRNs with the floral diversity in the surrounding landscape (Fig. 6a), collectively indicate a nuanced foraging strategy by *P. rothneyi* and underscores *P. rothneyi*'s ability to procure wood pulps from diverse resources. This adaptability allows them to establish nests successfully, regardless of the specific type of wood resources available in their immediate environment. It is crucial to note that the lower level of exclusive interaction observed in PMNs may be influenced by the relatively smaller number of studied PMNs. A more comprehensive analysis, involving additional samples from diverse localities, is imperative to establish a more robust comparison between the levels of taxa diversity and the interaction dynamics between *P. mandarinus* and the floral diversity in their surrounding landscapes. This approach will provide a more nuanced understanding of the ecological relationships shaping nest-building behaviors in different environments.

The collection of PRNs from Imha and ANU allowed for a comparison of the effect of land use on taxa richness and diversity between these localities. The Imha district is characterized by high proportion of natural plant habitat, whereas ANU has lower proportion of total vegetation and high proportion of non-plant habitat which observed in the landscape analysis of the 100 m radius around each nest (Supplementary Table 7). The higher taxa diversity and richness observed in PRNs collected from Imha can be attributed to the presence of more diverse plant taxa, especially woody plants, around the examined nests (Supplementary Table 9). While all ANU-collected PRNs are characterized by the high abundance of *Robinia*; *Salix* and *Pinus* are more abundant in Imha-collected PRNs, and *Robinia* was not detected in one of the Imha-collected nests (Fig. 1, Supplementary Table 6). In general, locations with higher vegetation cover are likely to support more diverse communities of plants, which can lead to higher taxa richness and diversity in wasp nest.

Except for *Quercus*, which is a forest species, most of the plant taxa collected by the wasps for nest construction were edge forest plants. Notably, all nests were collected under the roofs of buildings situated at the edge of the forests. The preference for edge forest plants in nest construction suggests that the wasps exhibit a strong affinity for plant species commonly found in the transition zones between forests and open areas. The presence of wasp nests under the roofs of buildings at the forest's edge highlights the adaptability of these wasps to human-altered environments. Understanding the wasps' selection of edge forest plants and their propensity to utilize anthropogenic structures can provide valuable insights into their ecological behavior and resource utilization strategies.

Nests of *P. rothneyi* characterized by their larger size and dark grayish color, which is remarkably different form *P. mandarinus* nests. Prior research generally confirms the effect of nesting material on the coloration of wasps’ nest. Production of blue nests by *D. sylvestris* and *D. norwegica* has been reported while providing a blue paper for nest construction^45^. Offering colorful papers at regular intervals resulted in the production of a colorful nest by *P. dominulus* (see Fig. 19.9 in Grasse^46^). Since all major taxa found in PRNs were also detectable in PMN samples, the difference in nest coloration cannot be attributed to the presence or absence of any plant taxa. The protein content and amino acid composition of the oral secretions of different wasp species are not similar^47,48^ and can be the source of dissimilarity in wasp nest coloration. The amount and composition of oral secretions involved in nest construction can be different in these two species. Incorporation of soil and sand in the nest construction of *V. crabro* has been documented^49^, which can be the possible source of darker coloration in *P. rothneyi* nests.

In conclusion, our study illuminates the intricate relationship between social wasps and plant resources, offering insights into their influence on nest-building material diversity and composition. Woody plants, notably *Quercus* and *Robinia*, emerge as crucial in this process. Our findings disclose preferences for specific plant resources, the impact of land use on resource availability, and potential factors like oral secretions shaping nest characteristics. These insights contribute to our understanding of social wasp ecology and behavior, with broader implications for insect-plant relationships in natural ecosystems. The DNA metabarcoding methodology, focused on specific *Polistes* species, holds potential applications for understanding floral choices in other *Polistes* and *Vespa* species, including invasive species like *Vespa velutina* and *V. mandarinia*. Recognizing the ecological requirements of these species is vital due to their potential impact on local ecosystems, especially in semi-urbanized areas. Insights into their preferred ecological conditions are essential for effective urban planning to mitigate potential threats. Regarding rearing practices, the common use of hard paper as a nesting material in controlled conditions can be naturalized to enhance ecological relevance and yield more realistic results, advancing our understanding of *Polistes* behavior. Furthermore, recognizing *Polistes* as edible insects presents an emerging trend, and our findings can improve rearing facilities, potentially contributing to sustainable food source development. Knowledge about essential plants for nest construction holds significance for conservation, particularly amid global warming challenges. Identifying and safeguarding specific plant species vital for nest construction allows for targeted conservation efforts, preserving Polistes populations and their ecological roles.

In summary, our study's implications extend beyond *Polistes* floral composition, offering valuable insights into broader ecological contexts, spanning invasive species management, urban planning, rearing practices, sustainable food sources, and proactive conservation. These multifaceted contributions enhance our understanding of complex ecological interactions.

## Materials and methods

### Preparation of known bark mixtures

Barks from nine woody plant species belonging to nine families were collected from a mixed-forest area near Andong, South Korea in 2021. Barks were only collected from the branches with 5cm diameter using a sterilized knife and were then transferred to the lab inside a plastic bag and kept at − 20 before being prepared into mixtures. Due to the high possibility of contamination by fungi, bacteria, or air-borne pollens, the barks were soaked in distilled water for 96 h, changing the water every 24 h. Afterward, they were transferred to a sterilized container, washed with absolute alcohol and a toothbrush, and then kept in an oven at 30 °C for 24 h. The dried bark samples were ground using liquid nitrogen and a mortar and pestle until they formed a fine powder which was used to prepare seven artificial bark mixtures. Plant species and the proportions of barks used in each mixture are listed in Table 1. Barks for each mixture were selected randomly, with each species present in at least three samples.

### DNA extraction, library preparation and sequencing

Bark mixtures were ground more for an additional 5 min using three 2 mm tungsten carbide beads. To extract the total DNA, 100 g of the bark mixtures were used, and a protocol developed for DNA isolation from bark^50^ was followed with some modifications. The extracted DNA was purified using the OneStep PCR Inhibitor Removal Kit (Zymo Research) and stored at −20 °C prior to subsequent analysis. We used a two-step PCR approach to amplify the *rbcL* and *trnL* barcode markers regions for library preparation. In the first round of PCR, we used universal primers with an Illumina overhanging adaptor. For the *rbcL* region, we applied *rbcL*2F^51^ and *rbcL* a-R^52^ universal primers, which have an amplicon length of approximately 500 bp. To amplify the *trnL* (UAA) intron, we used primers g and h^53^, which amplify a short fragment of this intron (10–143 bp) and can be useful for detecting plant species in old plant samples, such as bark, or in not very well-conserved material, such as wasps' nests. PCR was performed in a final volume of 25 μl, with 2.5 μl of template DNA combined with 12.5 μl of 2 × KAPA HiFi HotStart ReadyMix (KAPABIOSYSTEMS) and 5 μl of each primer (1 μM). The PCR cycle included an initial denaturing at 95 °C for 3 min, followed by 30 cycles of 95 °C for 30 s, 50 °C for 30 s, and 72 °C for 30 s, with a final extension at 72 °C for 5 min. The amplicons from the first PCR were purified using an AMPureXP purification kit, and 5 μl of the PCR product was used as a template in the second PCR. In a final volume of 50 μl, we used 25 μl of KAPA HiFi HotStart ReadyMix, 5 μl of Nextra XT Index Primer 1 (N7xx), 5 μl of Nextra XT Index Primer 2 (S5xx), and 10 μl of PCR Grade water. The PCR reaction was repeated as for the first PCR, but with 8 cycles. The second PCR was used to attach unique tags for separation of the sequences into samples after sequencing. The resulting products were purified using a AMPureXP purification kit. All PCR products were pooled in equal concentrations and sequenced in a single flow cell on a 600-cycle run of the Illumina MiSeq instrument (Illumina, USA) at Macrogen (South Korea). All sequence information has been deposited in the National Center for Biotechnology Information (NCBI) Sequence Read Archive (accession code PRJNA768940).

### Bioinformatic pipeline and taxonomic annotation

We utilized the bioinformatics pipeline developed by Mohamadzade Namin et al.^54^ to analyze the sequencing data. the demultiplexed reads with phred quality score below 25 were trimmed using Trimmomatic^55^, and only the reads with a length of more than 180bp for the *rbcL* marker and more than 10bp for *trnL* marker were retained. The forward and reverse reads were merged using VSEARCH, and the merged reads less than 450bp for *rbcL* marker and less than 10bp for *trnL* marker were discarded. The length-trimmed reads were then checked for chimeras using VSEARCH comparing to the *rbcL* and *trnL* reference databases created by OBITools3, which contained all relevant sequences downloaded from the EMBL database (2022.06.21), following the protocol outlined by Mohamadzade Namin et al.^54^. The chimera-free sequences were clustered at 97% threshold, and BLAST was used to assign taxonomy to the representative sequences. Reads were assigned to the species level when the top bit score matched with a single species, to the genus level when the top bit score matched with different species of the same genus, and to the family level when the top bit score matched with different genera belonging to the same family. Sequences that could not be identified at the family level were discarded. The scientific names of the Operational Taxonomic Units (OTUs) were compared to the list of Korean plants (http://koreanplant.info/wordpress/index.php/plantlist-eng/) and those with species name not available in the list were assigned to the genus level.

### Sequence count removal threshold

A negative control was utilized to determine sequence count removals, and the sequence count of each taxon from the negative control was set as a preliminary removal threshold for further analysis of the bark mixtures’ sequence reads. We then calculated the proportion of the sequences of each taxon and set the threshold of 0.01 proportion of reads for both molecular markers, based on the taxa available in the bark mixtures. We removed all sequence reads associated with taxa that were not present in the mixtures, as airborne pollen remaining on the bark surface after washing could be a potential source of contamination. Taxa with proportions lower than this threshold may not be reliably detectable. For the calculation of the median proportion, we only proceeded with taxa that were detected with both molecular markers. The median proportion of the floral composition of each nest was calculated using the following formula:

$${\text{Median}}\;{\text{proportion}}\, = \,\left( {{\text{Proportion}}\;{\text{A}}\;{\text{of}}\;{\text{taxon}}\, + \,{\text{Proportion}}\;{\text{B}}\;{\text{of}}\;{\text{taxon}}} \right)/{2}.$$

Proportion A: Proportion of each taxon using *rbcL* marker.

Proportion B: Proportion of the same taxon using *trnL* marker.

A limited number of false positive taxa were present in the bark mixtures, with a relative proportion of less than 0.0025, which we selected as a threshold for taxa removal in median analysis.

### Study the floral composition of wasps’ nest

#### Sample collection

Although many paper wasps hang their nests from tree branches, it can be challenging to locate these nests during summer when they are hidden by leaves. However, in fall, when leaves are removed from the trees, it becomes easier to find nests. Nonetheless, it is not always possible to attribute each nest to a wasp species. During the summer of 2020, nests of *Polistes* spp. were monitored, and thirteen nests of two different paper wasp species, *P. rothneyi* (8 nests) and *P. mandarinus* (5 nests), were collected in late September from two localities near Andong city, South Korea (Supplementary Fig. 1). Wasps from each colony were collected and identified to the species level using morphological identification. Nests were collected from the roofs or upper part of the window frames of buildings. Five *P. rothneyi* nests (PRNs) were collected from the northern edge of the Andong National University (ANU), where wasps had access to limited number of woody plants (Supplementary Table 5). Five *P. mandarinus* nests (PMNs) and three PRNs were collected from the roof of a historical school in the Imha district, which is surrounded by mixed forest area containing broadleaf and pine trees (Supplementary Table 5).

#### DNA extraction, and molecular analysis

After removing the maconia from the bottom of the cells, nests were washed using previously described method for bark sample preparation. The dried nests were then cut into small pieces using sterilized scissors and ground with a mortar and pestle using liquid nitrogen. DNA extraction, library preparation, sequencing, and bioinformatics pipelines were similar to those used for the bark mixture analysis. All sequence information has been deposited in the National Center for Biotechnology Information (NCBI) Sequence Read Archive (project number PRJNA999088).

#### Evaluation of vegetation cover in pulp foraging area

In order to explore how taxa richness and diversity are related to the vegetation cover, we assessed the amount of vegetation cover within the foraging range that surrounds each nest. The potential foraging range was defined as a circle with a 100-m radius centered on the nest, as previously determined by Prezoto and Gobbi^56^. We used landcover map data with 1 m spatial resolution from the Korea Ministry of Environment (KME) website (http://eng.me.go.kr/). A circle of 100m radius was given to the base map and the landcover types were extracted in ArcGIS pro^57^ and the total area of the vegetation (forest, grassland, agricultural farms) surrounding each nest were identified (Supplementary Table 5). Since the molecular identification of taxonomic units retrieved from the molecular analysis was conducted at the genus level, it is appropriate to calculate the richness and diversity of the surrounding area at the same taxonomic level. Therefore, the richness and diversity of the surrounding area were estimated at the genus level. The Chao1 formula^58^ was used to estimate the woody plant genus richness in the area, taking into account the number of observed genera, singletons (genera observed only once), and doubletons (genera observed exactly twice). Similarly, the Shannon–Wiener index^59^ was calculated to quantify the genus-level diversity within the area surrounding each nest collection point. The frequency or proportion of each genus was considered in the calculation, reflecting the relative abundance of different genera in the population.

$$Chao1={S}_{obs}+\frac{{a}^{2}}{2b}$$

where S_obs_ is the number of genus observed in the sample, a is the number of genus observed only once, b is the number of genus observed exactly twice.

$$\mathrm{Shannon }-\mathrm{ Wiener }=-{\sum }_{i=1}^{S}{{\uppi }_{i} {{\text{log}}}_{2}({\uppi }_{i})}$$

where S is the genus richness of the population, representing the total number of different, π_i_ is the frequency or proportion of the *i*th species in the population.

### Statistical analysis

We assessed the correlation between the proportion of DNA sequencing reads and the proportion of the artificial bark mixtures using a linear mixed-effects model (LMEM), implemented with the ImerTest package in R. Plant species within different mixtures were included as a random effect. This analysis was performed separately for each molecular marker and for the median proportion. The *rbcL* and *trnL* reads were converted into proportions. Due to the presence of false positives across bark mixtures, a threshold of 0.01 proportion for *rbcL* and *trnL* and 0.0025 for median proportion were applied and the taxa with a proportion lower than this level were removed before the LMEM analysis and the new proportion of the remaining taxa were recalculated for each molecular marker.

We used Spearman's rank correlation to assess whether each colony of wasps used the same plants in similar proportions in nest construction. Mann–Whitney U test was used to compare differences in taxa richness and Shannon diversity index between samples of each wasp species (*P. rothneyi* and *P. mandarinus*). As the PRN samples were collected from both localities, the effect of land cover on the taxa richness and diversity of Imha-collected PRNs and ANU-collected PRNs were also compared using Mann–Whitney U test. As the data did not meet the assumption of normality required for parametric tests, non-parametric tests were used. We also performed a regression analysis to assess the relationship between PRNs taxa richness and diversity with vegetation cover within the foraging range. The correlation between the proportion of plants observed in each nest was compared with the floral composition available in a 100-m radius landscape surrounding each nest using Spearman's rank correlation method. The objective was to understand the level of selectivity and adaptation exhibited by two wasp species in relation to the landscape. Furthermore, the regression analysis was performed to estimate the relatedness of the richness and diversity observed in the nest samples with the estimated richness and diversity of the surrounding area. Correspondence analysis (CA) was conducted using the "FactoMineR" package to visualize the relationships between plant taxa and their abundances in PRNs and PMNs. All statistical analyses were performed using R 4.1.0.

### Supplementary Information

Supplementary Information 1.

Supplementary Information 2.

Supplementary Information 3.

Supplementary Information 4.

## Data Availability

All sequence information has been deposited in the National Center for Biotechnology Information (NCBI) Sequence Read Archive (project number PRJNA999088).
