# Supplementary material for: Uncovering floral composition of paper wasp nests (Hymenoptera: Vespidae: Polistes) through DNA metabarcoding
Source: Sci Rep. 2024 Feb 3;14:2830. doi: 10.1038/s41598-024-52834-6 (PMC10838270; doi:10.1038/s41598-024-52834-6)
Supplement: Supplementary file 1 — Supplementary Information 1. [file 41598_2024_52834_MOESM1_ESM.docx]

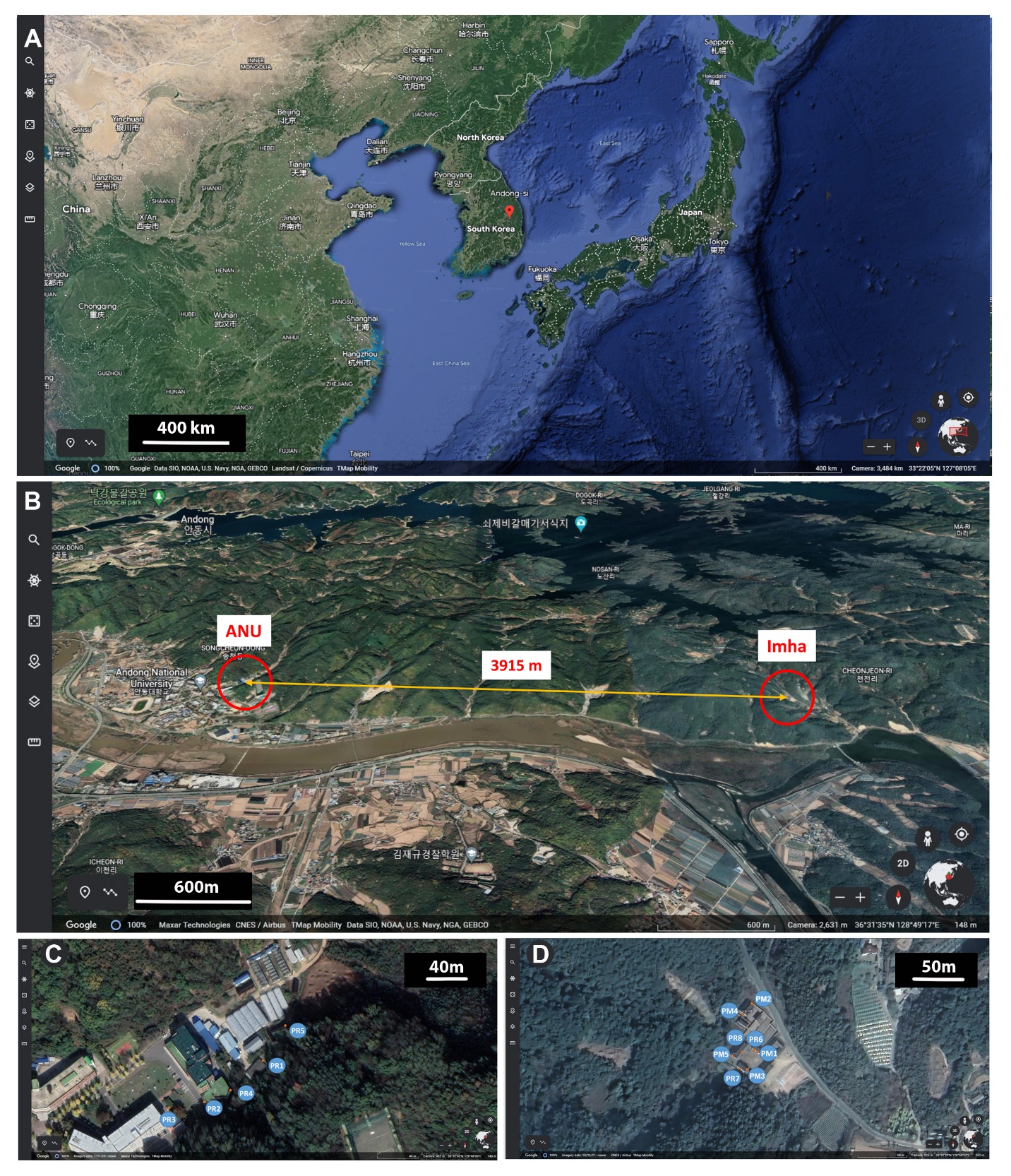


Supplementary figure 1. Google Earth satellite imagery depicting the geographical locations of the sites where nests of *P. mandarinus* (PM-n) and *P. rothneyi* (PR-n) were gathered for this study. Figure B illustrates the distance between the two collection sites, namely Andong National University (ANU) and Imha district. Figure C provides an enlarged view of the collection sites at ANU, while Figure D presents an enlarged view of the collection sites in Imha district.

Supplementary table 1. The number of trnL raw reads per bark mixture and the remaining reads after quality trimming, merging, length trimming, and chimera checking.

| Sample | Row reads | Quality trimmed | Merged reads | Length trimmed | Chimera free | Assigned taxonomy |
| --- | --- | --- | --- | --- | --- | --- |
| PC1 | 167786 | 164382 | 162313 | 162311 | 147058 | 144243 |
| PC2 | 184717 | 181511 | 179516 | 179516 | 178264 | 177552 |
| PC3 | 242298 | 237635 | 234647 | 234645 | 227146 | 225133 |
| PC4 | 186127 | 182296 | 175507 | 175507 | 166911 | 157975 |
| PC5 | 213508 | 209485 | 206596 | 206596 | 206348 | 205110 |
| PC6 | 202018 | 198296 | 195714 | 195713 | 193487 | 191756 |
| PC7 | 237047 | 232853 | 229931 | 229931 | 226560 | 220195 |
| NC (Negative control) | 137042 | 3320 | 3286 | 3286 | 3098 | 2652 |
| Total reads | 1570543 | 1409778 | 1387510 | 1387505 | 1348872 | 1324616 |

Supplementary table 2. The number of rbcL raw reads per bark mixture and the remaining reads after quality trimming, merging, length trimming, and chimera checking.

| Sample | Row reads | Quality trimmed | Merged reads | Length trimmed | Chimera free | Assigned taxonomy |
| --- | --- | --- | --- | --- | --- | --- |
| PC1 | 88785 | 70227 | 54922 | 54907 | 54527 | 54092 |
| PC2 | 110611 | 91270 | 78952 | 78951 | 77758 | 61059 |
| PC3 | 99737 | 82593 | 67862 | 67282 | 66172 | 63147 |
| PC4 | 70782 | 47529 | 31423 | 31400 | 29664 | 25797 |
| PC5 | 130098 | 107195 | 88193 | 87705 | 79394 | 79394 |
| PC6 | 92789 | 75268 | 59656 | 59652 | 56870 | 44707 |
| PC7 | 97113 | 78380 | 64541 | 64348 | 63093 | 52386 |
| NC (Negative control) | 44 | 0 | 0 | 0 | 0 | 0 |
| Total reads | 689959 | 552462 | 445549 | 444245 | 427478 | 380582 |

Supplementary table 3. Linear mixed effect model of the observed proportion of each taxa with the real proportion of the same taxa in seven bark mixtures.

| Parameter | Parameter estimate | SE | t | Model |
| --- | --- | --- | --- | --- |
| rbcL |  |  |  |  |
| Intercept | 0.064 | 0.0797 | 0.803 | R^2^=0.683  Adjusted R^2^=0.547  F=5.03  df= 21  p-value= 0.00109 |
| Real proportion | 0.67 | 0.192 | 3.499 |  |
| trnL |  |  |  |  |
| Intercept | 0.126 | 0.066 | 1.893 | R^2^=0.677  Adjusted R^2^=0.538  F=4.89  df= 21  p-value= 0.0013 |
| Real proportion | 0.412 | 0.152 | 2.698 |  |
| Median calculation |  |  |  |  |
| Intercept | 0.096 | 0.07 | 1.36 | R2=0.719  Adjusted R2=0.599  F=5.99  df= 21  p-value= 0.0003 |
| Real proportion | 0.538 | 0.155 | 3.456 |  |

Supplementary table 6. Plant taxa with the proportion higher than 0.01 in nests of *P. mandarinus* (PM-n) and *P. rothneyi* (PR-n), and the status of the recorded plants. The taxa richness and the shannon diversity index of each nest sample is given. Plant type: woody tree, shrub and vine W, herbaceous H.

| **Taxa** | **Plant type** | **ANU-collected nests** | | | | | **Imha-collected nests** | | | | | | | | |
| --- | --- | --- | --- | --- | --- | --- | --- | --- | --- | --- | --- | --- | --- | --- | --- |
|  |  | **PR-1** | **PR-2** | **PR-3** | **PR-4** | **PR-5** | **PR-6** | **PR-7** | **PR-8** | **PM-1** | **PM-2** | **PM-3** | **PM-4** | **PM-5** |  |
| ***Robinia pseudoacacia*** | **W** | 0.819435 | 0.739028 | 0.903707 | 0.667453 | 0.468321 |  | 0.31418 | 0.050175 | 0.728069 | 0.833184 | 0.193964 | 0.349476 | 0.030496 |  |
| ***Rosa*** | **W** |  |  |  | 0.037435 |  |  |  |  |  |  |  |  |  |  |
| ***Morus*** | **W** | 0.015831 |  |  | 0.023911 |  |  |  |  |  |  |  |  |  |  |
| ***Quercus*** | **W** | 0.100996 | 0.013284 | 0.057658 | 0.037409 | 0.517641 | 0.152171 | 0.020998 | 0.151293 | 0.180784 | 0.03861 | 0.03754 | 0.053187 | 0.251313 |  |
| ***Pinus*** | **W** |  | 0.039109 | 0.010027 |  |  | 0.548481 | 0.044949 |  |  |  |  | 0.016468 | 0.019441 |  |
| ***Ulmaceae*** | **W** |  |  |  |  | 0.014039 |  |  |  |  |  |  |  |  |  |
| ***Solanum*** | **W/H** | 0.048052 |  | 0.012981 |  |  |  |  | 0.028224 | 0.043505 | 0.017614 | 0.547636 |  |  |  |
| ***Chelidonium majus*** | **H** | 0.015687 |  |  |  |  |  |  |  |  |  |  |  |  |  |
| ***Salix*** | **W** |  | 0.010084 |  |  |  |  | 0.395979 | 0.24411 |  | 0.110592 | 0.187326 |  | 0.044473 |  |
| ***Oxalis*** | **H** |  |  |  |  |  |  |  | 0.012723 |  |  |  |  |  |  |
| ***Wisteria floribunda*** | **W** |  |  |  |  |  |  |  | 0.203513 |  |  |  |  |  |  |
| ***Humulus*** | **W** |  |  |  |  |  | 0.01001 |  |  |  |  |  |  |  |  |
| ***Prunus*** | **W** |  |  | 0.015627 |  |  |  | 0.205828 | 0.138046 | 0.036672 |  | 0.011792 | 0.015767 | 0.168198 |  |
| ***Cedrus deodara*** | **W** |  |  |  |  |  |  |  | 0.076512 |  |  |  |  |  |  |
| ***Sicyos*** | **H** |  |  |  |  |  |  |  |  | 0.01097 |  |  |  |  |  |
| ***Diospyros*** | **W** |  |  |  |  |  | 0.147919 |  |  |  |  | 0.021741 | 0.565103 | 0.48608 |  |
| ***Brassica*** | **H** |  |  |  |  |  | 0.105809 |  |  |  |  |  |  |  |  |
| ***Corylus*** | **W** |  |  |  |  |  | 0.021859 |  |  |  |  |  |  |  |  |
| ***Larix kaempferi*** | **W** |  | 0.198494 |  |  |  |  |  |  |  |  |  |  |  |  |
| ***Oryza*** | **H** |  |  |  |  |  | 0.013751 |  |  |  |  |  |  |  |  |
| ***Rosaceae*** | **W** |  |  |  |  |  |  | 0.018065 |  |  |  |  |  |  |  |
| ***Cucumis sativus*** | **H** |  |  |  |  |  |  |  | 0.095404 |  |  |  |  |  |  |
| ***Platanus*** | **W** |  |  |  | 0.233791 |  |  |  |  |  |  |  |  |  |  |
| **Taxa richness** | | **5** | **5** | **5** | **5** | **3** | **7** | **6** | **9** | **5** | **4** | **6** | **5** | **6** |  |
| **Taxa diversity** | | **0.671401** | **0.774988** | **0.423542** | **0.944787** | **0.75601** | **1.324871** | **1.349018** | **1.954481** | **0.847396** | **0.59236** | **1.220445** | **0.979043** | **1.319037** |  |

Supplementary table 7. Landcover composition of 100m radius around each nest collected from ANU and Imha region.

| Habitat | Type | Attribute | PR-1 | PR-2 | PR-3 | PR-4 | PR-5 | PR-6 | PR-7 | PR-8 | PM-1 | PM-2 | PM-3 | PM-4 | PM-5 |
| --- | --- | --- | --- | --- | --- | --- | --- | --- | --- | --- | --- | --- | --- | --- | --- |
| PM-5(Semi-) natural plant habitat | Natural | Grass land | 7.4 | 1.1 | 1.6 | 7.4 | 6.1 | 0.8 | 0.5 | 0.8 | 0.5 | 0.9 | 0.8 | 0.8 | 0.9 |
|  |  | Coniferous forests | 4.5 | 4.8 | 4.7 | 4.5 | 6.4 | 47.1 | 47.2 | 46.6 | 47.2 | 47.6 | 46.6 | 46.6 | 46.6 |
|  |  | Mixed forest | 0 | 0 | 0 | 0 | 0 | 0 | 0 | 0.9 | 0 | 0.91 | 0.9 | 0.9 | 0.9 |
|  |  | broadleaf forest | 18.2 | 19.3 | 19.1 | 18 | 24 | 43.5 | 43.6 | 43.1 | 43.6 | 41.9 | 43.1 | 43.1 | 43.1 |
|  | Agricultural crops | | 0 | 0 | 0.03 | 0 | 1.8 | 0 | 0 | 0 | 0 | 0 | 0 | 0 | 0 |
| Non-plant habitat | Natural | River | 0 | 0 | 0 | 0 | 0 | 0 | 0 | 0 | 0 | 0 | 0 | 0 | 0 |
|  | Disturbed | Facility | 0.7 | 1.3 | 1.5 | 1.3 | 0.6 | 0.01 | 0.01 | 0.01 | 0.01 | 0.01 | 0.01 | 0.01 | 0.01 |
|  |  | Residential area | 0 | 0 | 0 | 0 | 0 | 0.06 | 0.06 | 0.06 | 0.06 | 0.06 | 0.06 | 0.06 | 0.06 |
|  |  | Road | 68.7 | 73 | 72.5 | 68.3 | 60.5 | 7.8 | 7.8 | 7.8 | 7.9 | 8 | 7.8 | 7.8 | 7.7 |
|  |  | Bare land | 0.4 | 0.4 | 0.4 | 0.4 | 0.4 | 0.66 | 0.7 | 0.65 | 0.7 | 0.67 | 0.65 | 0.65 | 0.65 |
|  | | | |  |  |  |  |  |  |  |  |  |  |  |  |

Supplementary table 8. Regression model of the vegetation area around the nests of *Polistes rothneyi* and the taxa richness and taxa diversity of each nest.

| Parameter | Parameter estimate | SE | t | p | Model |
| --- | --- | --- | --- | --- | --- |
| Taxa richness | | | | | |
| Intercept | 3.442e+0 | 9.098e-1 | 3.783 | 0.00915 ** | R^2^=0.559  Adjusted R^2^=0.485  F=7.6  df= 6  p-value= 0.0329 |
| Vegetation area | 1.316e-04 | 4.771e-05 | 2.758 | 0.0329 * |  |
| Taxa diversity |  |  |  |  |  |
| Intercept | 3.115e-01 | 1.838e-1 | 1.69 | 0.14116 | R^2^=0.766  Adjusted R^2^=0.727  F=19.6  df= 6  p-value= 0.00443 |
| Vegetation area | 4.270e-05 | 9.641e-6 | 4.43 | 0.00443 ** |  |

Supplementary table 10. Regression model of the taxa richness and diversity inside *Polistes mandarinus* nests and the taxa richness and taxa diversity derived from 7 subsamples from 100m surrounding each nest.

| Parameter | Parameter estimate | SE | t | p | Model |
| --- | --- | --- | --- | --- | --- |
| Taxa richness of nest | | | | | |
| Intercept | 25.4743 | 6.8044 | 3.744 | 0.0333* | R^2^=0.01865  Adjusted R^2^=-0.3085  F=0.05702  df= 3  p-value= 0.8266 |
| Taxa richness of 100m landscape | -0.3093 | 1.2952 | -0.239 | 0.8266 |  |
| Taxa diversity of nest | |  |  |  |  |
| Intercept | 2.3518 | 0.1239 | 18.976 | 0.00032*** | R^2^=0.5885  Adjusted R^2^=0.4513  F=4.29  df= 3  p-value= 0.13.3 |
| Taxa diversity of 100m landscape | 0.2503 | 0.1209 | 2.071 | 0.1301 |  |

Supplementary table 11. Regression model of the taxa richness and diversity inside *Polistes rothneyi* nests and the taxa richness and taxa diversity derived from 7 subsamples from 100m surrounding each nest.

| Parameter | Parameter estimate | SE | t | p | Model |
| --- | --- | --- | --- | --- | --- |
| Taxa richness of nest | | | | | |
| Intercept | 25.6760 | 5.2867 | 4.857 | 0.00283 ** | R^2^=0.007687  Adjusted R^2^=-0.1577  F=0.04648  df= 6  p-value= 0.8364 |
| Taxa richness of 100m landscape | -0.1944 | 0.9017 | -0.216 | 0.83645 * |  |
| Taxa diversity of nest | |  |  |  |  |
| Intercept | 1.8541 | 0.1243 | 14.914 | 5.72e-06*** | R^2^=0.7225  Adjusted R^2^=0.6762  F=15.62  df= 6  p-value= 0.007518 |
| Taxa diversity of 100m landscape | 0.4375 | 0.1107 | 3.952 | 0.00752** |  |


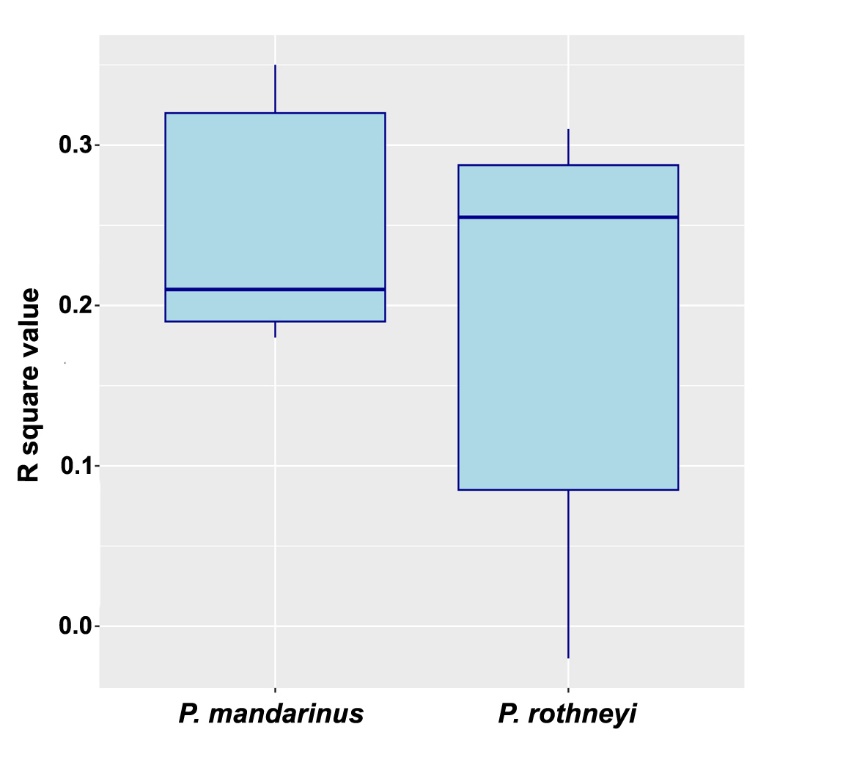


Supplementary figure 2. Box plot of the R square value of spearman rank correlation of the nest of each species with the taxa collected surrounding that nest.
